# Cadonilimab alone as neoadjuvant immunotherapy followed by radical resection for dMMR/MSI-H gastric adenocarcinoma: a case report

**DOI:** 10.3389/fimmu.2026.1774646

**Published:** 2026-09-21

**Authors:** Keqin Tan, Tingting Long, Hong Zhu

**Affiliations:** 1 Department of Medical Oncology, Cancer Center, West China Hospital, Sichuan University, Chengdu, Sichuan, China; 2 Department of Laboratory Medicine, West China Hospital, Sichuan University, Chengdu, China; 3 Clinical Laboratory Medicine Research Center, West China Hospital, Sichuan University, Chengdu, China

**Keywords:** cadonilimab, case report, deficient mismatch repair (dMMR), gastric adenocarcinoma, neoadjuvant immunotherapy

## Abstract

Gastric adenocarcinoma characterized by deficient mismatch repair (dMMR)/high microsatellite instability (MSI-H) represents a distinct subtype with high tumor immunogenicity and inherent sensitivity to immune checkpoint inhibitors. While neoadjuvant dual immunotherapy or chemoimmunotherapy has demonstrated high pathological complete response (pCR) rates in locally advanced dMMR/MSI-H gastric cancer, evidence supporting neoadjuvant monotherapy with Cadonilimab remains scarce. Cadonilimab is a PD-1/CTLA-4 bispecific antibody. We herein present the case of a 72-year-old man with locally advanced gastric antral adenocarcinoma (cT3N0M0, dMMR/MSI-H, PD-L1 CPS = 30) who received three cycles of neoadjuvant Cadonilimab (625 mg, Q3W) followed by radical surgery. Postoperative pathological assessment confirmed a pCR (TRG0), with no residual tumor identified in the gastric specimen or any of the 32 dissected lymph nodes. This case offers compelling evidence that Cadonilimab monotherapy can induce a profound pathological response with a favorable safety profile, supporting its further investigation as a personalized neoadjuvant strategy for this disease subset.

## Introduction

Gastric cancer (GC) remains a leading cause of global cancer-related morbidity and mortality, posing a severe threat to public health (1). Despite advancements in comprehensive treatment strategies centered on surgical resection, the prognosis of locally advanced gastric cancer (LAGC) remains suboptimal due to factors such as late diagnosis and high recurrence risk (2). In recent years, molecular subtyping has revolutionized the personalized treatment of GC, among which the deficient mismatch repair (dMMR)/high microsatellite instability (MSI-H) subtype has emerged as a distinct entity with unique biological characteristics (3, 4). Accounting for approximately 6-10% of gastric adenocarcinomas (5), dMMR/MSI-H tumors exhibit high immunogenicity driven by accumulated somatic mutations and neoantigen production, rendering them highly sensitive to immune checkpoint inhibitors (ICIs) (6, 7).

The paradigm of neoadjuvant therapy for resectable LAGC has evolved beyond conventional chemotherapy, with immunotherapy-based regimens showing promising efficacy (8). Clinical evidence indicates that neoadjuvant dual immune checkpoint blockade (such as nivolumab plus ipilimumab) or ICI combined with chemotherapy can achieve high pathological complete response (pCR) rates in dMMR/MSI-H LAGC (9–11), and may even enable non-surgical management in patients achieving clinical complete response (cCR) (12). However, the optimal neoadjuvant strategy for this subtype remains under investigation. Cadonilimab is a bispecific antibody that concurrently inhibits the PD-1 and CTLA-4 pathways, thereby exhibiting significant anti-tumor efficacy through dual immune checkpoint blockade. However, there remains a paucity of published evidence regarding its application as a standalone neoadjuvant treatment in patients with dMMR/MSI-H gastric adenocarcinoma (13).

PD-L1 expression, quantified by the CPS, serves as an important predictive biomarker for ICI efficacy (14). Patients with higher PD-L1 CPS typically exhibit more robust responses to immunotherapy, attributed to enhanced immune cell infiltration in the tumor microenvironment (15). For dMMR/MSI-H gastric cancer patients with high PD-L1 expression, neoadjuvant ICI monotherapy may offer a simplified, well-tolerated treatment option while maintaining substantial anti-tumor efficacy (16).

Against this backdrop, we report a case of dMMR/MSI-H locally advanced gastric antral adenocarcinoma with a high PD-L1 CPS (30) treated with neoadjuvant Cadonilimab monotherapy followed by radical surgery. This case aims to provide clinical evidence for the efficacy and safety of Cadonilimab monotherapy in this specific patient population, enrich the understanding of personalized neoadjuvant strategies for dMMR/MSI-H GC, and inform the management of similar cases in clinical practice.

## Case presentation

A schematic overview summarizing the patient’s treatment timeline is provided in **Figure 1**. The patient is a 72-year-old male with a 15-year history of well-controlled hypertension and a 1-year history of well-managed hyperlipidemia. The patient’s previous CT coronary angiography showed mild coronary atherosclerosis, with no history of chest pain, chest tightness, or other symptoms of angina. His father died of lung cancer, and no other immediate family members have a history of tumors.

**Figure 1:**
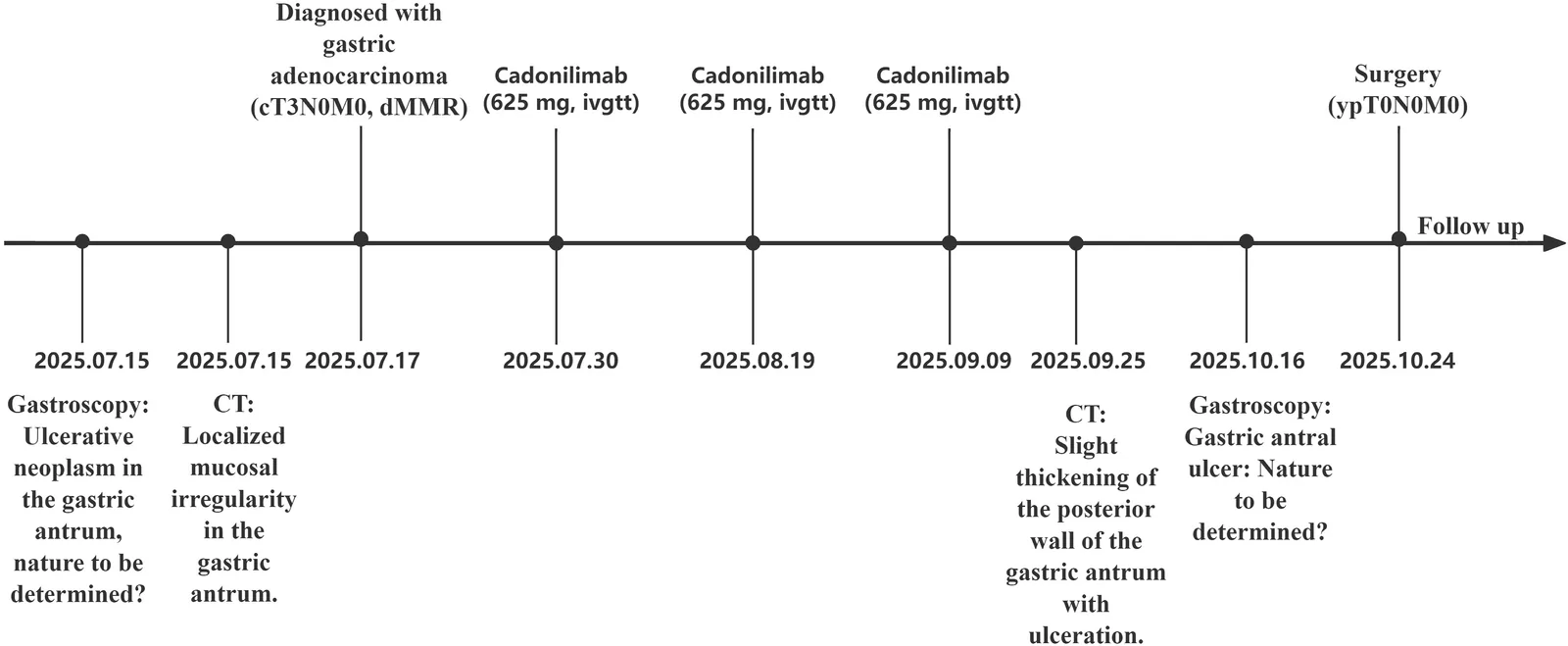
Overview of course of disease.

In July 2025, a gastroscopy performed during a routine physical examination revealed ulcerative neoplasms in the gastric antrum. A subsequent biopsy confirmed the diagnosis of gastric adenocarcinoma. Immunohistochemical profiling demonstrated a dMMR phenotype (MSH2+, MSH6+, PMS2-deficient), HER2 negativity (score 0), and Claudin18.2 expression (2+: 20%; 1+: 10%). PD-L1 22C3 testing revealed a CPS of 30 (TPS: 5%). *In situ* hybridization was negative for Epstein-Barr virus-encoded small RNA (EBER). Based on the dMMR profile, the tumor was classified as the MSI-H molecular subtype. The CT findings revealed localized mucosal irregularity in the gastric antrum, with relatively clear surrounding fat spaces and no obvious lymphadenopathy in the abdominal cavity or retroperitoneum (**Figure 2A**).

**Figure 2:**
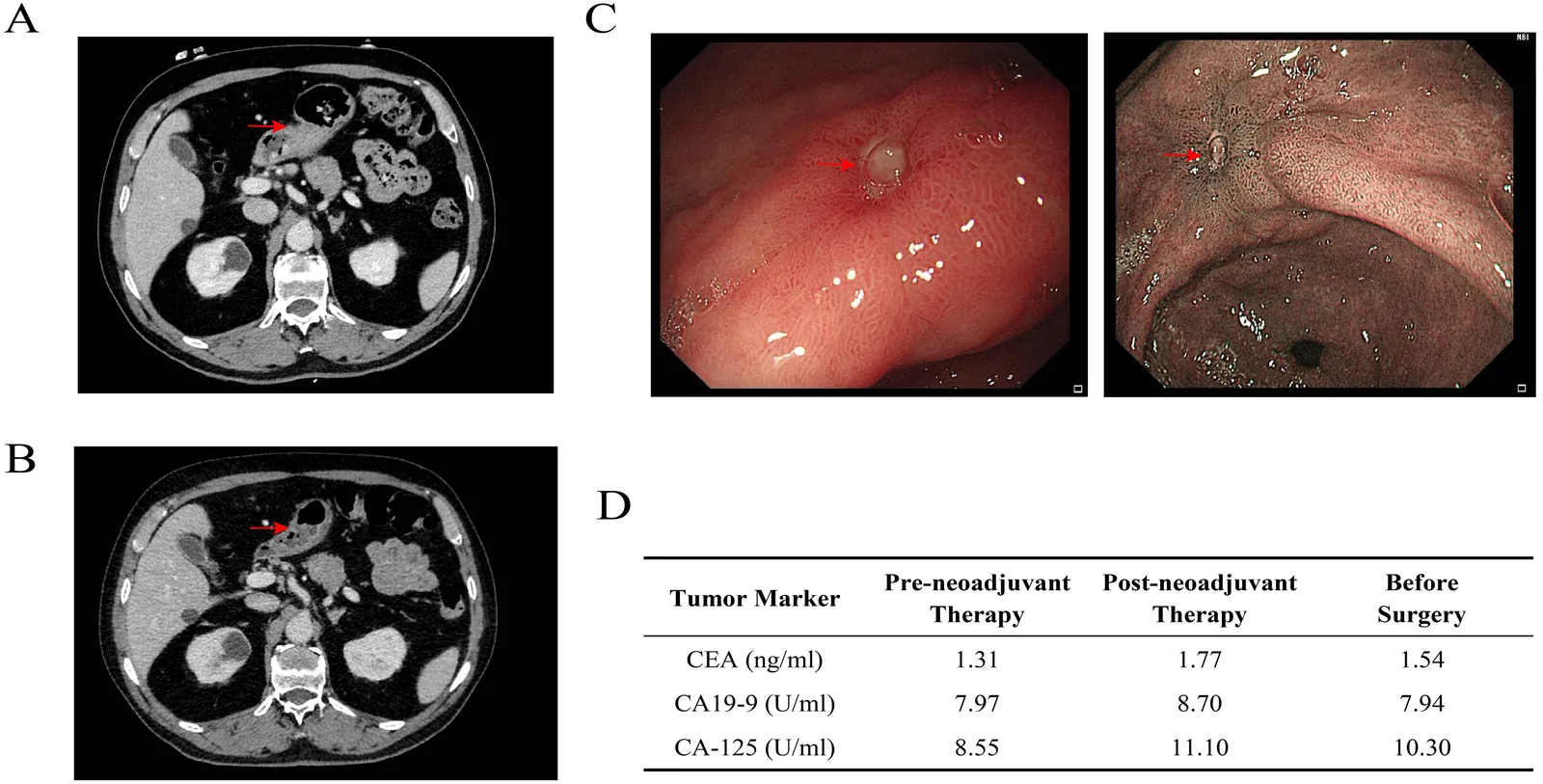
Imaging, endoscopic, and tumor marker changes during the patient’s diagnosis and treatment. The lesion site (gastric antral mucosal irregularity/slight thickening) is indicated with red arrows in panels **(A, B)**. **(A)** Pre-neoadjuvant therapy CT image, showing localized mucosal irregularity in the gastric antrum, clear surrounding fat spaces, and no enlarged lymph nodes; **(B)** Post-neoadjuvant therapy follow-up CT image, showing slight thickening of the posterior wall of the gastric antrum with a small mucosal depression, and no abnormalities in the serosal surface or surrounding fat spaces; **(C)** Post-neoadjuvant therapy follow-up gastroscopy image, indicating a 0.4 cm ulcer on the lesser curvature of the gastric antrum; **(D)** Changes in serum tumor marker levels (CEA, CA19-9, CA-125), showing a transient mild increase after treatment and a return to near baseline before surgery.

Between July 30 and September 9, 2025, the patient received three cycles of neoadjuvant therapy with Cadonilimab at our institution. The regimen consisted of Cadonilimab 625 mg administered intravenously on day 1 of each 21-day cycle. Treatment was well-tolerated, with no observed adverse reactions such as fever, rash, or diarrhea. Furthermore, no laboratory abnormalities of grade 1 or higher (as per CTCAE v5.0) were recorded in hepatic, renal, or thyroid function, or in myocardial enzyme profiles.

On September 25, 2025, the patient’s re-examination CT revealed poor gastric distension, slight local thickening of the posterior wall of the gastric antrum with a small depression on the mucosal surface, and clear serosal surface and surrounding fat spaces (**Figure 2B**). On October 17, 2025, a follow-up gastroscopy revealed a 0.4 cm ulcer on the lesser curvature of the gastric antrum and a 0.5 cm polypoid protrusion on the lesser curvature of the upper gastric body (**Figure 2C**). Biopsies from the antrum showed severe chronic inflammation with erosion, and from the gastric body moderate chronic inflammation with erosion; no neoplastic component was identified.

We performed a preoperative response assessment based on RECIST 1.1 and endoscopic findings. According to RECIST 1.1, the target lesion (gastric antral thickening) had reduced but still presented measurable residual abnormality, namely slight thickening with a small mucosal depression. Therefore, the best response was partial response (PR), not clinical complete response (cCR). Endoscopically, a 0.4 cm ulcer with surrounding mucosal edema persisted. Biopsy showed severe chronic inflammation without tumor cells. However, given the risk of sampling error and the lack of universally accepted endoscopic cCR criteria for gastric cancer, the patient was not considered to have achieved cCR. Consequently, a “watch-and-wait” strategy was not adopted.

The decision to proceed with radical surgery was supported by several considerations. First, regarding patient age and comorbidities, the 72-year-old patient with well-controlled hypertension and hyperlipidemia was deemed an acceptable surgical candidate. Second, the location of the tumor in the gastric antrum carries a non-negligible risk of lymph node metastasis, especially to stations 1, 3, 4sb, 4d, 5, 6 and others. Even with a good radiographic response, residual nodal disease could not be confidently excluded preoperatively. Third, unlike rectal cancer, there is no validated non-operative management protocol for gastric cancer. Endoscopic biopsy may miss residual tumor, and nodal status cannot be accurately determined without surgical sampling. Fourth, after thorough discussion, the patient and his family strongly favored radical resection to minimize recurrence risk. These points were incorporated into the clinical decision-making process.

Following comprehensive preoperative evaluation that excluded surgical contraindications, a laparoscopic-assisted radical distal gastrectomy with D2 lymphadenectomy and Billroth-II reconstruction was performed under general anesthesia on October 24, 2025. Intraoperative exploration identified no ascites, adhesions, or peritoneal implants (P0), and inspection of the liver, gallbladder, pancreas, spleen, and intestines revealed no metastatic disease. A 1.5 × 1.5 cm Borrmann type II ulcerated tumor was located in the gastric antrum, with no involvement of the esophagogastric junction. The tumor was mobile, showed no serosal invasion, and the operative field was without edema, exudate, or fibrosis. Proximal and distal resection margins were planned with a 5-cm safety margin from the tumor.

Intraoperative frozen section analysis of the duodenal margin, greater curvature margin, and lesser curvature margin of the stomach revealed no definite evidence of carcinoma involvement, a finding that was consistent with the subsequent permanent section diagnosis. Final postoperative pathology confirmed a pCR (TRG0). Microscopic examination showed only chronic mucosal inflammation with erosion, consistent with treatment effects, and no definitive residual carcinoma was identified on immunohistochemical staining. A total of 32 lymph nodes were dissected and examined from the following stations: group 1 (7 nodes), group 3 (3 nodes), group 4sb (5 nodes), group 4d (4 nodes), group 5 (5 nodes), group 6 (1 node), group 7 (1 node), group 8a (1 node), group 9 (1 node), group 11p (1 node), and group 12a (1 node). All lymph nodes were negative for metastasis.

During the patient’s neoadjuvant treatment with Cadonilimab, although serum tumor markers CEA, CA19-9, and CA125 consistently remained within the normal reference intervals, they exhibited a uniform dynamic pattern of a transient mild elevation post-treatment followed by a pre-surgical decline (**Figure 2D**). This trend of an initial rise succeeded by a fall, when considered alongside the patient’s ultimate achievement of a pathological complete response, strongly aligns with the characteristic biological signature of effective immunotherapy. The probable mechanism is that the immune checkpoint inhibitor successfully activated the host immune system, resulting in substantial tumor cell killing and the subsequent release of cellular contents into circulation, which in turn induced the observed transient fluctuation in marker levels. The key clinical and pathological findings before and after neoadjuvant therapy are summarized in **Table 1**.

**Table 1:** Summary of key clinical and pathological findings before and after neoadjuvant therapy.

Parameter	Pre-neoadjuvant (July 2025)	Post-neoadjuvant/Before surgery (September-October 2025)	Final outcome
Imaging (CT)	Localized mucosal irregularity in gastric antrum, clear fat spaces, no enlarged lymph nodes	Slight thickening of posterior antral wall with small mucosal depression, clear serosal surface and fat planes	Partial response (PR) per RECIST 1.1
Endoscopy & preoperative biopsy	Ulcerative neoplasm (initial diagnosis)	0.4 cm ulcer on lesser curvature of antrum; biopsy from antrum and gastric body showed severe chronic inflammation with erosion, no neoplastic cells	No residual tumor on pre-surgical biopsy
Tumor markers (CEA/CA19-9/CA-125)	CEA 1.31 ng/mL, CA19-9 7.97 U/mL, CA-125 8.55 U/mL	Transient mild elevation after second cycle: CEA 1.77, CA19-9 8.70, CA-125 11.10; then declined before surgery: CEA 1.54, CA19-9 7.94, CA-125 10.30. All values within normal ranges.	Characteristic fluctuation of effective immunotherapy
Postoperative pathology	–	–	pCR (TRG0), no residual carcinoma, 32 lymph nodes negative for metastasis

No adjuvant therapy was administered after surgery. The rationale follows. The patient achieved pCR (TRG0) with no residual tumor in the primary lesion or in any of the 32 lymph nodes. According to current guidelines (NCCN, CSCO), adjuvant therapy is not routinely recommended for dMMR/MSI-H gastric cancer patients who attain pCR after neoadjuvant immunotherapy. The potential benefit of additional therapy is uncertain, and the risks, including immune-related adverse events, outweigh any anticipated gain. The patient is under regular surveillance as per the discharge plan. The follow-up schedule is as follows: every 3 months during the first postoperative year, every 6 months during the second year, and then at progressively longer intervals thereafter. This schedule aligns with standard guidelines for advanced gastric cancer. At the 3-month follow-up, tumor markers and chest/abdominal CT showed no abnormalities.

As of June 2026, the patient has been followed for 7 months after surgery. Tumor markers (CEA, CA19-9, CA-125) have remained within normal limits, and chest/abdominal CT revealed no definite signs of recurrence or metastasis. The patient is generally well and has resumed normal daily activities.

## Discussion

This study reports a case of an elderly patient with dMMR/MSI-H locally advanced gastric adenocarcinoma who achieved a pathological complete response after just three cycles of neoadjuvant Cadonilimab monotherapy. This outcome not only confirms the high sensitivity of this molecular subtype to immunotherapy but, more importantly, reveals the substantial therapeutic potential of PD-1/CTLA-4 bispecific antibody monotherapy in this specific patient population.

The most significant finding in this case is that pCR was achieved through dual immune checkpoint blockade with a single agent, without the addition of chemotherapy. This offers a highly attractive, “chemotherapy-free” novel strategy for patients who cannot tolerate the toxicity of traditional chemotherapy or are considered chemotherapy-vulnerable due to advanced age or comorbidities.

The choice of cadonilimab is supported by its mechanism as a PD-1/CTLA-4 bispecific antibody that simultaneously blocks two immune checkpoints. In the highly immunogenic dMMR/MSI-H tumor microenvironment, this dual blockade may provide a stronger synergistic anti-tumor effect than a single-agent PD-1 inhibitor (17). Moreover, compared to combining two separate monoclonal antibodies such as nivolumab plus ipilimumab, cadonilimab offers the advantage of single-agent administration, potentially reducing infusion burden and additive toxicity. Other PD-1 inhibitors including pembrolizumab and nivolumab have shown activity in dMMR/MSI-H gastric cancer, but head-to-head comparisons are lacking. This case provides initial real-world evidence supporting further investigation of cadonilimab, and future randomized trials are needed to compare different ICI strategies.

The excellent safety profile observed during treatment is equally noteworthy. The absence of any significant immune-related adverse events ensured that the subsequent radical surgery could proceed as planned without perioperative complications. This compares favorably with the higher toxicity profiles typically linked to conventional dual immunotherapy combinations such as nivolumab plus ipilimumab. Cadonilimab’s unique pharmacological properties, which may drive localized immune activation and reduced systemic toxicity, could contribute to its improved therapeutic window. This attribute is crucial for patients undergoing major surgery.

Furthermore, the high PD-L1 expression (CPS = 30) in this case may have accurately identified a subgroup of patients most likely to benefit from this intensive immunotherapy regimen. This suggests that future personalized treatment decisions could be based on a more refined stratification, incorporating not only dMMR/MSI-H status but also PD-L1 expression and potentially other biomarkers like tumor mutational burden (TMB).

It is worth briefly comparing our case with the experience in dMMR rectal cancer, where a “watch-and-wait” strategy has been successfully explored. Gastric cancer, however, carries a higher risk of lymph node metastasis, and current evidence does not support routine non-operative management for gastric cancer, even in dMMR/MSI-H subtypes. Therefore, radical surgery remained the standard of care in this patient, who did not achieve a clinical complete response.

From the patient’s perspective, the 72-year-old patient was concerned about chemotherapy-related toxicities, including nausea, vomiting and alopecia. After learning that his locally advanced tumor harbored specific molecular features (dMMR/MSI-H, high PD-L1), he discussed with his physician and opted for a chemotherapy-free approach with cadonilimab monotherapy. Preoperative re-evaluation showed no residual tumor components on gastroscopic biopsy, a pleasant surprise for the patient. With good postoperative recovery, he is gradually returning to normal daily life.

Naturally, as a single case report, these conclusions require validation in large-scale clinical trials. Several critical questions remain unanswered, such as the optimal number of neoadjuvant cycles, the necessity of adjuvant therapy after achieving pCR, and the long-term survival benefits. Although the follow-up duration is only 7 months, which is insufficient to draw definitive conclusions about long-term survival or recurrence, the patient remains disease-free to date. The patient continues to be followed regularly, and long-term outcomes will be reported in future updates.

In summary, this case strongly demonstrates that neoadjuvant Cadonilimab monotherapy is a novel approach for dMMR/MSI-H locally advanced gastric cancer, characterized by remarkable efficacy and a favorable safety profile. It significantly enriches the therapeutic options in this field and provides valuable evidence for advancing precision immunotherapy towards more deeply personalized practice.

## Data Availability

The original contributions presented in the study are included in the article/supplementary material. Further inquiries can be directed to the corresponding author.
